# Tuberculose extrapulmonaire multi résistante: à propos de 7 cas

**DOI:** 10.11604/pamj.2019.32.196.17995

**Published:** 2019-04-23

**Authors:** Hanane Haddaoui, Fatima Zahra Mrabet, Mohammed Aharmim, Jamal-Eddine Bourkadi

**Affiliations:** 1 Service de Pneumo-Phtisiologie, Hôpital Moulay Youssef, CHU Rabat, Akkari, Faculté de Médecine et de Pharmacie de Rabat, Maroc

**Keywords:** Tuberculose, multirésistante, extra-pulmonaire, delanamid, Tuberculosis, resisting, extrapulmonary, delamanid

## Abstract

La tuberculose multi-résistante constitue un défi majeur pour l’Organisation Mondiale de la Santé. Son incidence croissante, les difficultés diagnostiques et thérapeutiques en font un problème de santé publique surtout dans les pays en développement. Nous rapportons deux cas de tuberculose résistante extra pulmonaire exclusive (ganglionnaire et ostéo-articulaire) et cinq cas associant à l’atteinte pulmonaire d’autres localisations extra pulmonaires (pleurale, ganglionnaire, anale et neuro-méningée) chez des patients hospitalisés à l’hôpital Moulay Youssef de Rabat. Ces cas illustrent la problématique de la résistance aux antituberculeux et soulèvent le rôle des tests génotypiques dans le diagnostic de la tuberculose extra-pulmonaire.

## Abstract (en)

Multidrug-resistant tuberculosis is a major challenge for the World Health Organization. Its growing incidence and the diagnostic and therapeutic difficulties make it a public health problem especially in the developing countries. We report two cases of extrapulmonary drug-resistant tuberculosis (nodal and osteo-articular) and five cases of pulmonary tuberculosis and extra-pulmonary tuberculosis (pleural, nodal, anal and neuro-meningeal) in hospitalized patients at the Hopital Moulay Youssef in Rabat. This study reports the issue of drug-resistant TB and highlights the role of genotypic tests in the diagnosis of extrapulmonary tuberculosis.

## Introduction

L’émergence de la tuberculose (TB) à bacilles résistants aux antituberculeux compromet le contrôle mondial de la tuberculose. Les souches multi résistantes (MDR), c’est-à-dire résistantes à l’isoniazide et à la rifampicine, sont apparues dans les années 90. Parmi ces souches, 6% ont développés des mécanismes de résistance supplémentaires aux principaux antituberculeux de deuxième ligne à savoir les fluoroquinolones et les aminosides. Ces souches sont dites ultrarésistantes (XDR). Sur le plan diagnostique, les tests génotypiques permettent de faire un diagnostic rapide des résistances aux antituberculeux en quelques heures. Il est désormais recommandé de réaliser un diagnostic génotypique de résistance à la rifampicine pour tout nouveau cas de tuberculose. Le pronostic de ces tuberculoses est sombre compte tenu du peu de ressources thérapeutiques disponibles. Toutefois, de nouveaux antituberculeux comme la bédaquiline et le délamanide offrent des perspectives d’amélioration du traitement et permettent d’envisager un changement radical dans la prise en charge [1]. Les tuberculoses extrapulmonaires (TPE) représentent un pourcentage croissant de toutes les formes de tuberculose, atteignant 20 à 40% d’entre elles selon les séries [2]. Les pays du Maghreb enregistrent les taux les plus élevés de TPE dans le monde ≈ 45 à 60%. À notre connaissance, il n'existe pas de données de littérature sur la prévalence de la tuberculose extra-pulmonaire résistante aux antibacillaires, et seuls quelques cas sont signalés. Nous rapportons deux cas de tuberculose résistante extra pulmonaire exclusive (ganglionnaire et ostéo-articulaire) et cinq cas associant à l’atteinte pulmonaire d’autres localisations extra pulmonaires (pleurale, ganglionnaire, anale et neuro-méningée) chez des patients hospitalisés à l’hôpital Moulay Youssef de Rabat. Ces cas illustrent la problématique de la résistance aux antituberculeux et soulèvent le rôle des tests génotypiques dans le diagnostic de la tuberculose extra-pulmonaire.

## Patient et observation

### Observation 1

**Figure 1:**
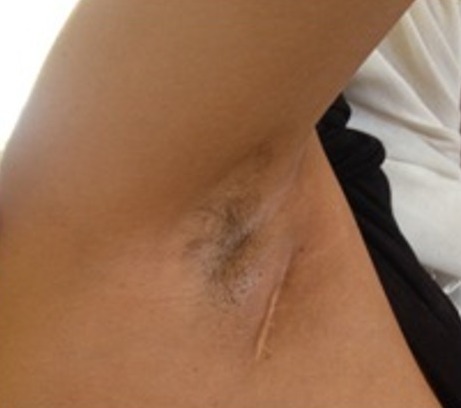
Cicatrice du curage ganglionnaire axillaire après 3 mois de traitement

Il s’agit d’une femme de 42 ans, ayant comme antécédent une tuberculose ganglionnaire cervicale droite traitée il y a 14 ans. L’histoire de sa maladie remonte à une année par l’apparition d’une tuméfaction axillaire droite augmentant progressivement de volume, inflammatoire et qui s’est fistulisée spontanément sans signes respiratoires associés évoluant dans un contexte de conservation de l’état général. L’examen clinique à l’admission était sans particularité à part celui des aires ganglionnaires qui retrouvait une adénopathie axillaire droite fistulisée (Figure 1). Le diagnostic de tuberculose ganglionnaire a été retenu par le Genexpert réalisé sur un fragment de biopsie qui a permis de détecter le mycobactériumtuberculosis (MT) et de confirmer la résistance à la rifampicine, l’étude anatomopathologique a révélé la présence d’un tissu inflammatoire granulomateux non caséeux. Le test GenoType MTBDRplus a objectivé une résistance à la rifampicine et une résistance de haut niveau à l’isoniazide. Le test GenoType MTBDRplus a montré une résistance de haut niveau à l’Ofloxacine ainsi qu’à l’amikacine et à la kanamycine. Ces deux tests ont permis de retenir le diagnostic de tuberculose ganglionnaire ultrarésistante XDR. La radiographie thoracique était normale, la recherche de bacilles acido-alcoolo-résistants (BAAR) dans les expectorations était négative. Le bilan biologique d’admission était sans particularité notamment la sérologie VIH était négative.

### Observation 2

**Figure 2:**
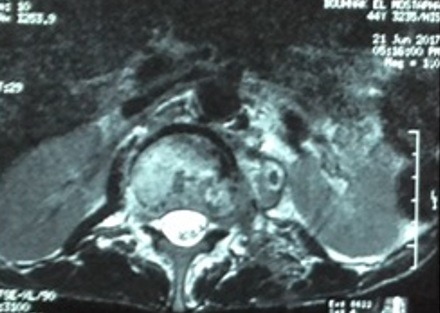
IRM du 21/06/2017: spondylodiscite L1-L2 avec collections au niveau des parties molles

**Figure 3:**
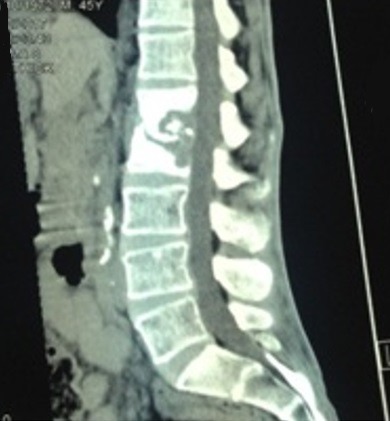
TDM dorso lombaire du 11/10/2017: lyse osseuse en miroir des corps L1 et L2 avec destruction totale du disque intervertébral et du mur postérieur ainsi qu’un envahissement du canal lombaire et extension de la collection sur toute l’étendue du psoas gauche

Il s’agit d’un patient de 45 ans, tabagique chronique 20 paquets/années sevré, mis sous traitement antibacillaire depuis mai 2016 pour une pleurésie tuberculeuse. L’évolution était marquée par l’apparition 3 mois après d’une paraparésie des membres inférieurs; une imagerie par résonance magnétique (IRM) dorso lombaire (Figure 2) a objectivé une spondylodiscite L1-L2; une biopsie vertébrale a été effectuée qui a révélé un granulome épithélio-giganto-cellulaire centré focalement de nécrose caséeuse; malheureusement l’étude bactériologique et les tests de sensibilité n’ont pas été réalisés. La décision était de poursuivre le traitement antibacillaire mais l’évolution était marquée par l’aggravation de la symptomatologie sur le plan clinique et radiologique, le scanner dorsolombaire de contrôle (Figure 3) a montré une spondylodiscite L1-L2 avec apparition de collections abcédées des parties molles (muscle psoas et muscles para vertébraux). Devant cette aggravation, le patient a bénéficié d’une ponction de l’abcès du psoas, le Genexpert réalisé sur le liquide de cytoponction a permis de détecter le MTB avec résistance à la rifampicine. Le test GenoType MTBDR plus a objectivé une résistance de haut niveau à l’isoniazide et une résistance à la rifampicine. Le test GenoType MTBDR sl a montré une résistance de haut niveau à l’ofloxacine et une sensibilité à l’amikacine et à la kanamycine. Par ailleurs, le patient était asymptomatique sur le plan respiratoire. La radiographie du thorax était normale. La recherche de BAAR dans les expectorations est revenue négative. Le bilan biologique était sans particularité, la sérologie VIH est négative. On a ainsi retenu le diagnostic d’une spondylodiscite tuberculeuse (Mal de Pott) pré XDR avec résistance haut niveau aux quinolones.

### Observation 3

Il s’agit d’une patiente de 56 ans ayant comme antécédent une tuberculose pleurale traitée en 2013. La symptomatologie remonte à 6 mois par l’installation d’une hémoptysie de faible abondance évoluant dans un contexte d’altération de l’état général. L’examen clinique à l’admission a révélé une adénopathie axillaire droite inflammatoire non fistulisée. La radiographie du thorax a montré des opacités micronodulaires et nodulaires bilatérales confluentes par endroit dont certaines sont calcifiés avec attraction de la trachée et coiffe pleurale bilatérale. La recherche de BAAR dans les expectorations est revenue positive à l’examen direct (ED) et le Genexpert a détecté le MT avec résistance à la rifampicine. Le test GenoType MTBDRplus a montré une résistance de haut niveau à la rifampicine et à l’isoniazide. Le test GenoType MTBDRplus a montré une résistance de haut niveau à l’ofloxacine et était sensible à la kanamycine et à l’amikacine. Une cytoponction de l’adénopathie a été réalisée et la culture a mis en évidence des BAAR. Le bilan biologique et la sérologie VIH était normaux. On a ainsi retenu le diagnostic d’une tuberculose pulmonaire pré XDR associée à une atteinte ganglionnaire.

### Observation 4

**Figure 4:**
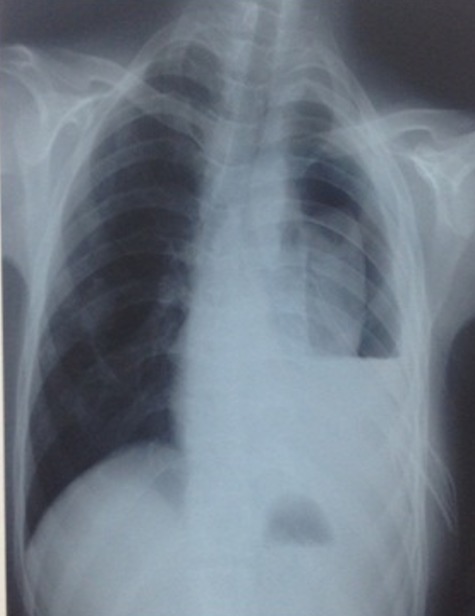
Pyopneumothorax gauche

**Figure 5:**
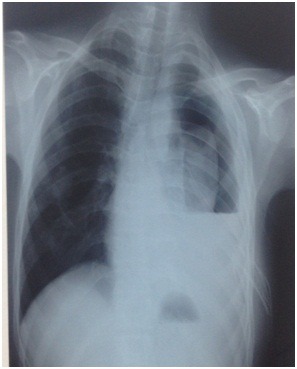
Pyopneumothorax gauche après drainage

Il s’agit d’un patient de 26 ans, tabagique chronique à 40 paquets/années, suivi pour tuberculose pulmonaire confirmée par examen bactériologique des expectorations. Il a rapporté 1 mois après le début du traitement, l’installation de douleurs basithoraciques droites de type pleurale. L’exploration radiologique a montré une pleurésie droite dont la ponction biopsie pleurale était en faveur d’une pleurite tuberculeuse et le diagnostic d’une réaction paradoxale a été retenu. Le bilan biologique a montré une anémie hypochrome microcytaire et la sérologie VIH était négative. Cependant, l’évolution était marquée par la négativation des BAAR du 2^ème^ mois de traitement et l’apparition d’un pyopneumothorax drainé (Figure 4 et Figure 5) sans amélioration d’où son hospitalisation au service de chirurgie thoracique pour décortication et pleurectomie. L’exploration chirurgicale a révélé 3 lobes atélectasiés avec présence de caséum et de petites cavités remplis de pus; une culture et un test Genotype MTBDR plus ont été réalisé sur le prélèvement du caséum qui ont montré des BAAR avec résistance à la rifampicine et à l’isoniazide. Le Genotype MTBDRplus n’a pas été réalisé car n’était pas encore disponible au Maroc. On a ainsi retenu le diagnostic d’une tuberculose pulmonaire multirésistante (TB MDR) avec atteinte pleurale associée.

### Observation 5

Il s’agit d’un patient de 49 ans ayant comme antécédent un pyopneumothorax tuberculeux traité en 2008 et admis actuellement pour une tuberculose pulmonaire confirmée bactériologiquement. L’évolution 2 mois après le début du traitement était marquée par l’apparition d’un épanchement pleural gauche et sur le plan bactériologique par la positivité des BAAR à l’ED, le Genexpert sur expectoration a montré une résistance à la rifampicine. Le Genotype MTBDRsl n’a pas été réalisé car n’était pas encore disponible au Maroc. Une ponction biopsie pleurale était en faveur d’une pleurite tuberculeuse. Le bilan biologique était sans particularité, la sérologie VIH était négative. On avait ainsi retenu le diagnostic d’une tuberculose pulmonaire multirésistante MDR associée à une atteinte pleurale.

### Observation 6

**Figure 6:**
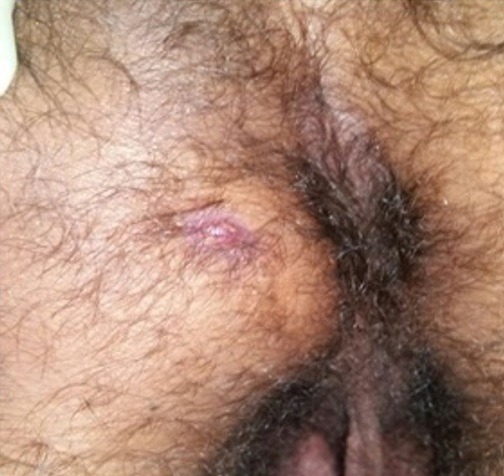
Fistule anale

Patient de 40 ans, tabagique chronique sevré, ayant comme antécédent une tuberculose pulmonaire confirmé bactériologiquement traitée à deux reprises en 2008 et en 2010. Il est revenu en 2017 dans un tableau de toux productive avec anorexie et amaigrissement, par ailleurs le patient rapporte des douleurs anales lancinantes avec écoulement de pus. L’examen pleuropulmonaire retrouvait des râles ronflants bilatéraux et des crépitants au niveau des bases. L’examen proctologique à l’admission retrouvait un abcès anal avec un orifice purulent visible à 2 centimètres de la marge anale (Figure 6). La radiographie du thorax a objectivé des opacités nodulaires et micronodulaires diffuses bilatérales des deux hémi champs thoracique avec attraction de la trachée. La recherche de BAAR dans les expectorations est revenue positive à l’examen direct (ED) et le Genexpert a détecté le MT avec résistance à la rifampicine. Le test GenoType MTBDRplus a montré une résistance de haut niveau à la rifampicine et une sensibilité à l’isoniazide. Le test GenoType MTBDRsl a montré une résistance à l’ofloxacine et était sensible à la kanamycine et à l’amikacine. Une ponction de la zone abcédée a permis l’issu de pus franc et l’examen bactériologique a mis en évidence la présence de bacilles acido-alcoolorésistants (BAAR) à l’examen direct. On a ainsi retenu le diagnostic d’une tuberculose pulmonaire pré XDR avec atteinte anale associée.

### Observation 7

Il s’agit d’une patiente de 33 ans, originaire du Gabon, suivie pour une infection rétrovirale, qui rapporte, une toux chronique avec des céphalées intenses et sensation de vertige. L’examen neurologique retrouve une patiente, mutique, un GCS à 10 au dépend de la réponse verbale et motrice, apyrétique, une nuque raide, la marche et la station debout sont impossibles, le reste de l’examen neurologique ne révélait pas d’autres anomalies. La radiographie du thorax a montré un discret syndrome bronchique péri-hilaire sans foyer alvéolaire ni formation nodulaire suspecte. Un Genexpert dans les expectorations a été réalisé et a permis de détecter le MTB avec résistance à la rifampicine. Une TDM cérébrale est revenue sans anomalie. Une ponction lombaire a montré un aspect rosâtre clair en goutte, l’étude cytobactériologique a montré un liquide céphalorachidien à prédominance lymphocytaire et le Genexpert a permis la détection du MT avec résistance à la rifampicine. Les tests GenoType MTBDR n’ont pas été réalisés par manque de disponibilité. Le bilan biologique retrouvait un syndrome inflammatoire biologique (protéine C réactive: 136mg/L), la numération des lymphocytes CD4 était à 184 cellules/mm^3^. On a ainsi retenu le diagnostic d’une tuberculose pulmonaire multirésistante MDR associée à une atteinte méningée sur terrain d’immunodépression sévère.

## Discussion

L’émergence de la tuberculose à bacilles résistants (TB-MR ou XDR-TB) est venue accroître la menace portée sur les progrès réalisés dans la maîtrise de la tuberculose [3]. La tuberculose multirésistante (MDR) est une infection à mycobactéries résistantes à l’isoniazide et la rifampicine, les 2 médicaments majeurs du traitement de la tuberculose. Parmi les tuberculoses MDR, on distingue les tuberculoses « pré ultra-résistante » (pré-XDR) quand les mycobactéries sont également résistantes aux fluoroquinolones ou à un antituberculeux injectable de deuxième ligne et « ultra-résistante » (XDR) lorsque les mycobactéries sont résistantes à ces quatre classes thérapeutiques [4]. La TB résistante survenant dans les sites extra pulmonaires est très rare en raison de la rareté des bacilles. Il s’agit essentiellement d’un problème d’origine humaine dû à un traitement inadéquat ou mal administré. Elle survient parfois chez un patient sans antécédents de traitement antituberculeux en raison d'une infection par un individu hébergeant des bacilles résistants [5]. L’OMS estime qu’il y a dans le monde, 630.000 cas de TB-MR, soit 5,3% du total des cas prévalents. Près de 60% de ces cas sont retrouvés en Inde, en Chine et en Fédération de Russie. On estime que parmi ces cas de tuberculose multi-résistante, 6% sont en fait des tuberculoses ultrarésistantes.

En Afrique, l’estimation porte sur environ 69.000 cas, dont la très grande majorité serait méconnue à cause des capacités insuffisantes des laboratoires à mettre en place des techniques de culture de M. tuberculosis et des tests de sensibilité aux antibiotiques, et aussi à cause de l’accès limité aux médicaments de seconde ligne [6]. Au Maroc, 30.651 cas de tuberculose, toutes formes confondues, ont été enregistré en 2017, la tuberculose extra pulmonaire représente 48% des cas, 236 cas de TB MR et 13 cas XDR ont été confirmés au laboratoire. Les jeunes, âgés de 15 à 45 représentent 64% des cas, le sex-ratio masculin/féminin est de 1,5. Grace au progrès en matière de lutte antituberculeuse au Maroc, la prévalence de la TB multi-résistante est maintenue à un niveau très bas: 1% de résistance primaire et 8,7% de résistance secondaire [7]. Les signes et les symptômes évocateurs de TB-MR ou XDR sont semblables à ceux des patients atteints de TB pharmacosensible. Cependant, du fait qu’elle est diagnostiquée tardivement, la TB multirésistante peut avoir une présentation clinique et radiologique plus sévère. Le test Genexpert a été réalisé chez tous nos patients (1 fois sur un fragment de biopsie ganglionnaire, 1 fois sur l’abcès du psoas, 1 fois sur le prélèvement pulmonaire chirurgical, 1 fois sur le LCR et 4 fois sur les expectorations) et a permis de détecter le MTB avec résistance à la rifampicine dans tous les cas. Les tests de MTBDRplus et sl n’ont été réalisés que chez 4 de nos patients (patients 1, 2, 3, 6) et 1 de nos patient (patient 4) n’a bénéficié que du MTBDRplus. La résistance à la rifampicine est presque toujours causée par une mutation survenant dans le gène rpoB. Les mutations peuvent être détectées en séquençant le gène rpoB ou en utilisant des dosages moléculaires commerciaux (Xpert^®^MTB / RIF, GenoTypeMTBDRplus^®^, INNOLIPA Rif-TB^®^). Ces techniques rechercheront la présence des mutations les plus fréquemment observées parmi les souches résistantes à la rifampicine, elles permettent un diagnostic rapide, en quelques heures seulement, et leurs utilisation hiérarchisée, permet de réduire le délai de diagnostic de la pharmacorésistance antituberculeuse. Selon les études, la sensibilité du GeneXpert est plus importante dans les fragments de biopsies, pus, LCR et liquides d’aspirations gastriques (sensibilité > 80%) que dans les liquides des séreuses (sensibilité < 50%) [8]. Certains des tests disponibles aident à détecter les mutations associées à la résistance à l'isoniazide (GenoTypeMTBDRplus^®^) ou à la résistance aux médicaments antituberculeux de deuxième intention (GenoTypeMTBDRsl^®^).

Ces tests sont associés à une excellente efficacité dans la détection de la résistance à la rifampicine (gène rpoB), avec une bonne efficacité pour l'isoniazide (gènes inhA et katG) [1]. Deux évaluations de la bandelette GenoType^®^ MTBDRslont été publiées. Les résultats obtenus sont bons mais imparfaits pour les fluoroquinolones et l’amikacine (sensibilité ≈ 90%), moins bons pour la kanamycine et la capréomycine (sensibilité: ≈ 80%) et insuffisants pour l’éthambutol (sensibilité: ≈ 60%).Il n’existe pas de bandelettes pour la détection moléculaire de la résistance au pyrazinamide qui repose sur le séquençage du gène pncA [9]. L’émergence et la transmission de la TB résistante aux médicaments, y compris la TB ultrarésistante (XDR), sont dues en partie à la relation synergique entre TB et VIH. Selon des données probantes, les personnes infectées par le VIH risquent davantage de souffrir de TB-XDR [10]. Le VIH favorise aussi les rechutes de tuberculose et la mortalité est accrue si le diagnostic n’est pas fait précocement [11]. Le Maroc est un pays à faible prévalence pour le VIH et la prévalence de la co-infection TB-VIH est de 1,7% [12].

En Afrique du Sud, d’après une étude menée par Neel R *et al*, les taux de co-infection par le VIH dépassaient 70% chez les cas de TB MDR et XDR alors que seulement 15 et 22% des patients atteints de tuberculose MDR et XDR, recevaient un traitement antirétroviral avant leur diagnostic de tuberculose pharmacorésistante [13]. Dans notre série, une seule patiente (Patiente 7) était suivie pour une infection rétrovirale diagnostiquée avant la tuberculose multirésistante. Les protocoles prescrits étaient divers associant (levofloxacine, kanamycine, ethionamide, cycloserine, linezolide, clofazimine, pyrazinamide, ethambutol, isoniazide forte dose et delamanide) respectant tous les bases de prescription de l’OMS. Selon nos connaissances, aucune donnée clinique n’est disponible sur l’utilisation du delamanide pour traiter la tuberculose extra-pulmonaire [14]. Le Tableau 1 récapitule les différents protocoles administrés à nos patients en fonction de leurs profils de résistances. L'Organisation mondiale de la santé (OMS) a mis à jour la classification des nouveaux médicaments antituberculeux 2016 (Tableau 2). Chez les patients atteints de tuberculose résistante à la rifampicine ou multirésistante, un traitement comportant au moins cinq antituberculeux efficaces pendant la phase intensive est recommandé, il comprend le pyrazinamide et quatre antituberculeux de deuxième intention, un du groupe A, un du groupe B et au moins deux du groupe C. Si le minimum de médicaments antituberculeux efficaces ne peut être composé, un agent du groupe D2 et d'autres agents du groupe D3 peuvent être ajouté pour porter le total à cinq [14].

**Tableau 1:** Protocoles administrés en fonction des profils de résistances

	Profil de résistance	Schéma thérapeutique
Patient 1	H, R,Ofx, Am, Km	Pto,lzd, cfz, Z, E, Dlm
Patient 2	H, R, Ofx	Amk, Pto, Lzd, Cfz, Z, E,Dlm
Patient 3	H,R, Ofx	Lfx, Km, Pto, Cs,Z, E, Vit B6
Patient 4	H, R	Lfx, Km, Pto, Cs, Z, E, Vit B6
Patient 5	R	Lfx, Km,Pto, Cs, Z, E, Vit B6
Patient 6	H, R, Ofx	Km, Lzd, Cfz, Z, H(h), Dlm
Patient 7	R	Lfx, Km, Pto, Cs, Z, E,H(h), Vit B6

H: Isoniazide, H(h) : Isoniazide forte dose, R: Rifampicine, Z: Pyrazinamide, E: Ethambutol, Amk : Amikacine, Km: Kanamycine, Pto: Prothionamide, Cs : Cyclosérine, Dlm: Délamanide, Lzd: Linézolide, Lfx: lévofloxacine, Cfz : Clofazimine, Vit B6 : Vitamine B6.

**Tableau 2:** Classification de l’Organisation Mondiale de la Santé 2016 des antituberculeux de 2^ème^ ligne

A. Fluoroquinolones	Levofloxacine Moxifloxacine Gatifloxacine.
B. Second-line injectable agents	Amikacine Capreomycine Kanamycine (Streptomycine).
C. Other core second-line agents	Ethionamide / Prothionamide Cyclosérine / Terizidone Linezolide Clofazimine.
D. Add-on agents	D1: Pyrazinqmid Ethambutol High-dose isoniazid
	D2: Bedaquiline Delamanid
	D3: Acideaminosalicylique, Imipénem-cilastatine, Amoxicilline-clavulanate (Thioacétazone)

Le traitement comporte deux phases: une première phase dite d’attaque et une seconde phase d’entretien. Le traitement d’attaque comporte quatre à six substances dont l’efficacité est certaine. En cas de traitement empirique, avant le résultat définitif de l’antibiogramme, le protocole doit comprendre au moins trois antituberculeux que le patient n’a jamais reçus. Pour ne pas risquer de sélectionner des résistances en cascade, il ne faut jamais ajouter un médicament seul à un protocole thérapeutique inefficace [15]. Les médicaments de deuxième ligne sont généralement largement distribués dans la plupart des fluides corporels et des tissus [14]. La pyrazinamide et la cyclosérine traversent la barrière hémato-encéphalique même en l’absence d’inflammation méningée, tandis que l’éthambutol et la streptomycine le font seulement en présence d’une inflammation méningée [2]. D’après l’OMS, le delamanide peut avoir un intérêt particulier chez les patients atteints de tuberculose MDR et qui présentent un risque plus élevé de mauvais résultats thérapeutiques (exemple: intolérance ou contre-indication, maladie extensive ou avancée), une résistance additionnelle aux fluoroquinolones ou aux antituberculeux injectables et une TB-XDR [4]. Dans notre étude, la sensibilité aux médicaments de deuxième ligne a été recherchée uniquement pour l'ofloxacine et la kanamycine. Malheureusement, aucun test de sensibilité des autres médicaments de deuxième ligne n'était disponible, ce qui aurait pu entraîner une administration inutile de médicaments inefficaces. Cela nécessite la mise en place d'infrastructures et de laboratoires dotés des dernières technologies pour tester la sensibilité aux autres médicaments [16]. Chaque patient a été suivi mensuellement pour l'évaluation clinique (poids corporel), l'examen de la culture des expectorations, la radiographie thoracique, un bilan hématologique et biochimique systématique ainsi qu’un ECG en cas de prise de délamanide et la surveillance des effets indésirables jusqu'à achèvement de la durée du traitement. Nous n’avons pas relevé d’effets secondaire majeurs nécessitant l’arrêt ou le changement du protocole thérapeutique, par ailleurs des effets secondaires mineurs ont été observés (intolérance digestive chez 3 patients, prurit chez un patient, perturbation modérée du bilan hépatique chez 2 patients) améliorés par simple traitement symptomatique.

**Figure 7:**
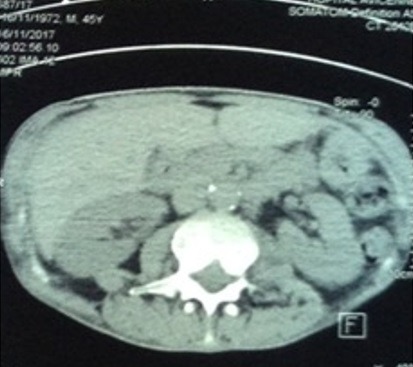
TDM dorso lombaire de contrôle 16/11/2017 montrant une petite lésion de 1cm du psoas gauche

L’évolution sous traitement était favorable chez 6 de nos patients marquée par une prise de poids de 3kg et une cicatrisation complète de la fistule (Patiente 1), une récupération fonctionnelle satisfaisante après plusieurs séances de rééducation et une régression importante de la collection purulente à la TDM dorso lombaire de contrôle (patient 2) (Figure 7), une négativation des BAAR à l’examen direct au premier mois de traitement pour le patient 3, un début de cicatrisation de la plaie et une diminution de la quantité du pus provenant de l’orifice du drainage post opératoire après 1 mois de traitement (patient 4), une prise de poids de 2 kg et une régression de l’abcès avec cicatrisation de la fissure anale au 2^ème^ mois de traitement (Patient 6). On déplore le décès de la patiente 7 au 3^ème^ mois de traitement en raison de la sévérité de l’atteinte méningée. Le pronostic de la TB-MR s'est amélioré au cours des dernières années grâce à la création d'équipes spécialisées, la mise en place de protocoles thérapeutiques personnalisés, et la prescription de nouveaux antibiotiques actifs contre Mycobacterium tuberculosis comme le linézolide et la bédaquiline [1]. Dans une analyse rétrospective des cas français de TB-MR par exemple, le taux de succès du traitement à deux ans était de 41,5%. Le pronostic des patients présentant une TB XDR est beaucoup moins optimiste, avec des taux de mortalité atteignant 50% à 100% [1]. Le succès thérapeutique est cependant toujours lié au nombre d'antibiotiques efficaces encore disponibles pour traiter les patients [1]. Par ailleurs, il n’existe pas de données actuelles ou d’études montrant l’évolution des patients sous traitement anti bacillaire de deuxième ligne pour une tuberculose extra pulmonaire résistante.

## Conclusion

La prévalence de la tuberculose résistante augmente ces dernières années en raison de la sensibilisation accrue à la maladie, de l'accès à la culture et des tests de sensibilité aux médicaments et de la suspicion précoce de cas de tuberculose résistante parmi les patients précédemment traités. On s'attend donc à ce que la tuberculose résistante affectant les sites extrapulmonaires augmente dans un proche avenir. Par conséquent, il y a un besoin urgent de sensibiliser davantage les médecins à une telle présentation [17].

## Conflits d’intérêts

Les auteurs ne déclarent aucun conflit d'intérêts.

## References

[1] Maitre T, Aubry A, Jarlier V, Robert J, Veziris N, CNR-MyRMA (2017). Multidrug and extensively drug-resistant tuberculosis. Med Mal Infect.

[2] Mazza-Stalder J, Nicod L, Janssens JP (2012). Extrapulmonary tuberculosis. Rev Mal Respir.

[3] N'guessan K, Bakayoko AS, Ahui-Brou JM, Kouakou AO, Coulibaly B, Guei A, Dosso M (2015). Extensively drug-resistant Mycobacterium tuberculosis strains isolated in Côte d'Ivoire. Med Mal Infect.

[4] Haute Autorité de Santé Commission de la transparence.

[5] Das SK, Das A, Gangopadhyay A, Sinha AK (2012). Primary disseminated extrapulmonary multidrug resistant tuberculosis. Indian J Med Microbiol.

[6] Tritar F, Daghfous H, Ben Saad S, Slim-Saidi L (2015). Management of multidrug-resistant tuberculosis. Rev Pneumol Clin.

[7] Ministère de la santé du Maroc (2018). Brochure progrès défis de la Tuberculose.

[8] Sylvie Audrey Diop, Aminata Massaly, Daye Ka, Noel Magloire Manga, Louise Fortes-Déguénonvo, Cheikh Tidiane Ndour (2016). Utilisation du test GeneXpert pour le diagnostic de la tuberculose au service des maladies infectieuses du CHNU de Fann. The Pan African Medical Journal.

[9] Truffot-Pernot C, Veziris N (2011). Bacteriological tests for tuberculosis. Rev Mal Respir.

[10] Alexander PE, De P (2007). The emergence of extensively drug-resistant tuberculosis (TB): TB/HIV coinfection, multidrug-resistant TB and the resulting public health threat from extensively drug-resistant TB, globally and in Canada. Can J Infect Dis Med Microbiol.

[11] Fuhrman C, Veziris N, Herer B, Bonnaud F (2004). Quelles attitudes adopter devant des séquelles de tuberculose mises en évidence sur une radiographie thoracique?. Rev Mal Respir.

[12] Docplayer (2013-2016). Plan national d'accélération de la réduction de l'incidence de la tuberculose.

[13] Gandhi NR, Shah NS, Andrews JR, Vella V, Moll AP, Scott M (2010). HIV coinfection in multidrug- and extensively drug-resistant tuberculosis results in high early mortality. Am J Respir Crit Care Med.

[14] Simon Tiberi, Ruaridh Buchanan, José Caminero A, Rosella Centis, Marcos Abdo Arbex, Miguel Salazar (2017). The challenge of the new tuberculosis drugs. Presse Med.

[15] Fréchet-Jachym M, Métivier N (2009). Tuberculose résistante. EMC (Elsevier Masson SAS, Paris), Pneumologie.

[16] Prajapati K, Mishra V, Desai M, Solanki R, Naik P (2017). Treatment outcome of patients having extensively drug-resistant tuberculosis in Gujarat, India. Int J Mycobacteriol.

[17] Mittal N, Bansal P (2014). Multidrug resistant extrapulmonary tuberculosis - three case reports and review of literature. Intern Med Inside.

